# Enzymatic Characterization of a Human Acyltransferase Activity

**DOI:** 10.1371/journal.pone.0005426

**Published:** 2009-05-04

**Authors:** Akihiko Ozawa, Richard B. Speaker, Iris Lindberg

**Affiliations:** Department of Anatomy and Neurobiology, University of Maryland—Baltimore, Baltimore, Maryland, United States of America; University of Oulu, Finland

## Abstract

**Background:**

Non-histone protein acylation is increasingly recognized as an important posttranslational modification, but little is known as to the biochemical properties of protein serine acylating enzymes.

**Methodology/Principal Findings:**

We here report that we have identified a metal-stimulated serine octanoyltransferase activity in microsomes from human erythroleukemic (HEL) cells. The HEL acylating enzyme was linear with respect to time and protein, exhibited a neutral pH optimum (stimulated by cobalt and zinc), and inhibited by chelating reagents. Hydroxylamine treatment removed most, but not all, of the attached radioactivity. A salt extract of microsomal membranes contained the major portion of enzyme activity, indicating that this acyltransferase is not an integral membrane protein. Sucrose density fractionation showed that the acyltransferase activity is concentrated in the endoplasmic reticulum. In competition experiments, the acyltransferase was well inhibited by activated forms of fatty acids containing at least eight to fourteen carbons, but not by acetyl CoA. The zinc-stimulated HEL acyltransferase did not octanoylate proenkephalin, proopiomelanocortin, His-tagged proghrelin, or proghrelin lacking the amino-terminal His-tag stub of Gly-Ala-Met. The peptides des-acyl ghrelin and ACTH were also not acylated; however, des-acyl ghrelin containing the N-terminal tripeptide Gly-Ala-Met was acylated. Mutagenesis studies indicated a requirement for serine five residues from the amino terminus, reminiscent of myristoyl transferase, but not of ghrelin acylation. However, recombinant myristoyl transferase could not recapitulate the hydroxylamine sensitivity, zinc-stimulation, nor EDTA inhibition obtained with HEL acyltransferase, properties preserved in the HEL cell enzyme purified through four sequential chromatographic steps.

**Conclusions/Significance:**

In conclusion, our data demonstrate the presence of a zinc-stimulated acyltransferase activity concentrated in the endoplasmic reticulum in HEL cells which is likely to contribute to medium-chain protein lipidation.

## Introduction

Protein acylation is increasingly recognized as critical for the regulation of a wide variety of cellular processes, including gene expression, protein localization, and intracellular signaling. The most frequently acylated residues within proteins are lysines (as in histone N-acetylation) and cysteines (as in protein S-palmitoylation); serines seem to be seldom acylated. However, certain bioactive peptides, such as α-MSH and β–endorphin, are known to exist in serine-acetylated forms [1]. The gastric peptide ghrelin is also serine-acylated, a modification which contributes greatly to its bioactivity (reviewed in [2], [3]). Serine and threonines within specific effector proteins are acetylated by the plague bacterial enzyme YopJ, a modification involved in pathogenicity [4]. Novel secretory pathway lysine acylating enzymes which contribute to protein targeting have also recently been demonstrated [5]. It therefore seems likely that other as-yet undiscovered protein posttranslational processing enzymes exist.

In this study, we present the biochemical characterization of a protein serine acyltransferase present in the microsomal fraction of HEL cells, which we have termed ERAT (endoplasmic reticulum O-acyl transferase). While we have used a modified proghrelin as a substrate, as described below, this HEL enzyme is enzymatically distinct from the modifying enzyme ghrelin O-acyl transferase (GOAT) [6], [7], and most likely physiologically acylates proteins other than proghrelin.

## Results

### The Microsomal Fraction of HEL Cells Contains Acyltransferase Activity

**Figure 1:**
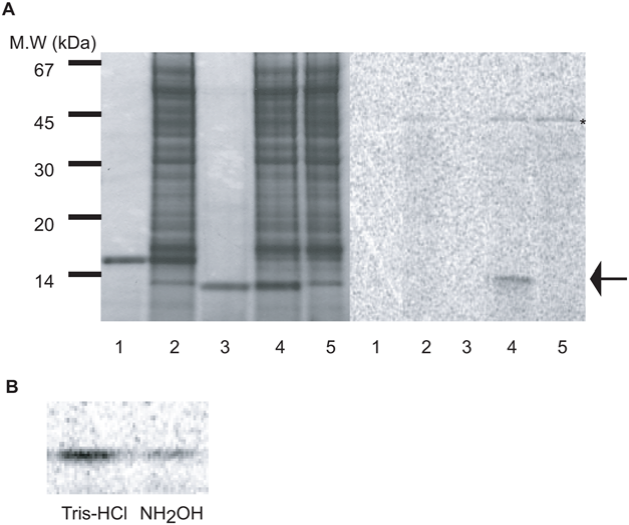
The microsomal fraction of HEL cells contains acyltransferase activity. (A) Acyltransferase activity was tested using [^14^C]octanoic acid transfer to either His-tagged proghrelin or GAM-proghrelin and the P2 microsomal fraction from HEL cells as an enzyme source. The reactions were carried out under the standard reaction conditions at 37 C for 2 h and then analyzed on 16.5% polyacrylamide gels. *Left panel*, Coomassie-stained gel of reaction mixtures to demonstrate the presence of equal quantities of His-tagged proghrelin and GAM-proghrelin; *right panel*, autoradiogram of the same reaction mixtures to identify the [^14^C]octanoylated band. *Lane 1,* His-tagged proghrelin alone*; lane 2,* His-tagged proghrelin with P2 microsomal fraction*; lane 3,* GAM-proghrelin*; lane 4,* GAM-proghrelin with P2 microsomal fraction*; lane 5,* P2 microsomal fraction alone. An arrow and asterisk indicate [^14^C]octanoylated GAM-proghrelin and endogenous substrate protein, respectively. (B) The removal of [^14^C]octanoic acid from [^14^C]octanoylated GAM-proghrelin was performed using either 1 M Tris-HCl, pH 8.0 or 1 M NH_2_OH, pH 8.0 (B).

Our initial interest was in identifying an enzyme capable of acylating proghrelin. Since substantial quantities of acylated ghrelin are made in a human erythroleukemia (HEL) cell line [8], [9], and since HEL cell ghrelin is acylated to a much higher extent than stomach ghrelin [8], we reasoned that HEL cells must possess enzymatic activity capable of transferring fatty acids to ghrelin. A microsomal extract was prepared from HEL cells and acyltransferase activity was examined by following the transfer of [^14^C]octanoic acid from [^14^C]octanoyl CoA to a bacterially-expressed modified proghrelin, followed by phosphorimaging. Figure 1A, left panel, shows the profile of Coomassie-stained proteins present in the reaction mixtures, while the right panel, a phosphorimage of the same gel, shows that only a modified proghrelin protein bearing the Gly-Ala-Met (GAM) amino-terminal tripeptide remaining from TEV (tobacco etch virus) protease cleavage can serve as a substrate for the reaction; amino-terminally His-tagged proghrelin was inactive as a substrate (compare lanes 2 and 4). No [^14^C]octanoyl CoA transfer to Gly-Ala-Met- proghrelin (GAM-proghrelin) occurred in the absence of an enzyme source (compare lanes 3 and 4). Boiling prior to inclusion in the assay also totally eliminated the ability of the HEL microsomal fraction to transfer octanoate (data not shown). The radioactive signal could be mostly, though not entirely removed from GAM-proghrelin by hydroxylamine treatment (Figure 1B), indicating the probable existence of an ester bond between octanoate and GAM-proghrelin. Since others have shown that hydroxylamine treatment effectively removes acyl groups from serine-acylated proghrelin (ref. 7, Yang *et al*.), the fact that a consistent amount of radioactive GAM-proghrelin persisted after overnight hydroxylamine treatment may indicate the presence of a small amount of N-acyltransferase activity, which would generate an amide bond resistant to hydroxylamine. In sum, these assays clearly demonstrate the presence of acyltransferase activity in HEL cell microsomes.

Interestingly, we consistently detected another octanoylated protein of approximately 46 kDa (Figure 1A; see asterisk). Because reactions lacking the substrate GAM-ghrelin also exhibit this octanoylated band (see lane 5), the HEL cell microsomal fraction must contribute this endogenous substrate protein.

**Figure 2:**
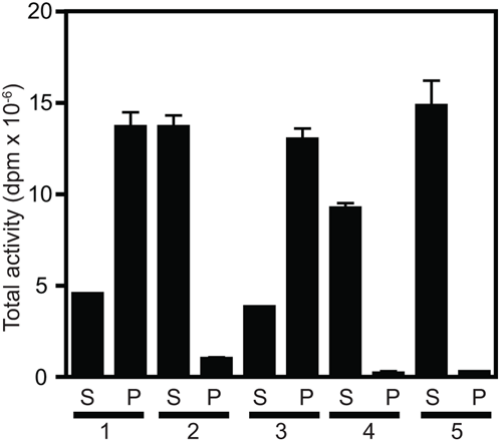
Acyltransferase activity can be extracted from HEL cell microsomes with high salt. Proteins in the P2 pellet were extracted according to the procedures described in Materials and Methods, dialyzed, and assayed for enzyme activity under the standard reaction conditions. Proteins were extracted under the following conditions: *Set 1,* using 10 mM Tris-HCl; *set 2*, 1 M NaCl; *set 3*, 1% Triton X-100; *set 4,* 1% sequential extraction first with Triton X-100, then the pellet extracted with 1 M NaCl; *set 5*, simultaneous extraction with 1% Triton X-100 and 1 M NaCl. *S*, supernatant; *P*, pellet. Results are given as dpm of total octanoyltransferase activity (reaction dpm multiplied by the total protein in each fraction). Samples were assayed in duplicate and the mean and standard deviation are shown.

In order to determine whether the human acyltransferase is membrane-bound or soluble, microsomal membranes were extracted with 1 M NaCl, 1% Triton X-100, or both. Data in Figure 2 demonstrate that treatment of microsomal membranes with 1 M NaCl extracted approximately 75% of enzyme activity, while 1% Triton X-100 did not solubilize the enzyme (Figure 2, reaction sets 1–3). When salt and detergent were combined, 80% of enzyme activity was recovered in the soluble fraction (Figure 2, set 5). Similarly, 80% of the enzyme activity could be extracted from the Triton-insoluble fraction using 1 M NaCl (Figure 2, set 4). These data show that the majority of acyltransferase activity is membrane-associated but is not integral to membranes. Further, this acyltransferase clearly distributes to Triton-resistant membranes.

### Acyltransferase Activity Is Localized to the Endoplasmic Reticulum (ER)

**Figure 3:**
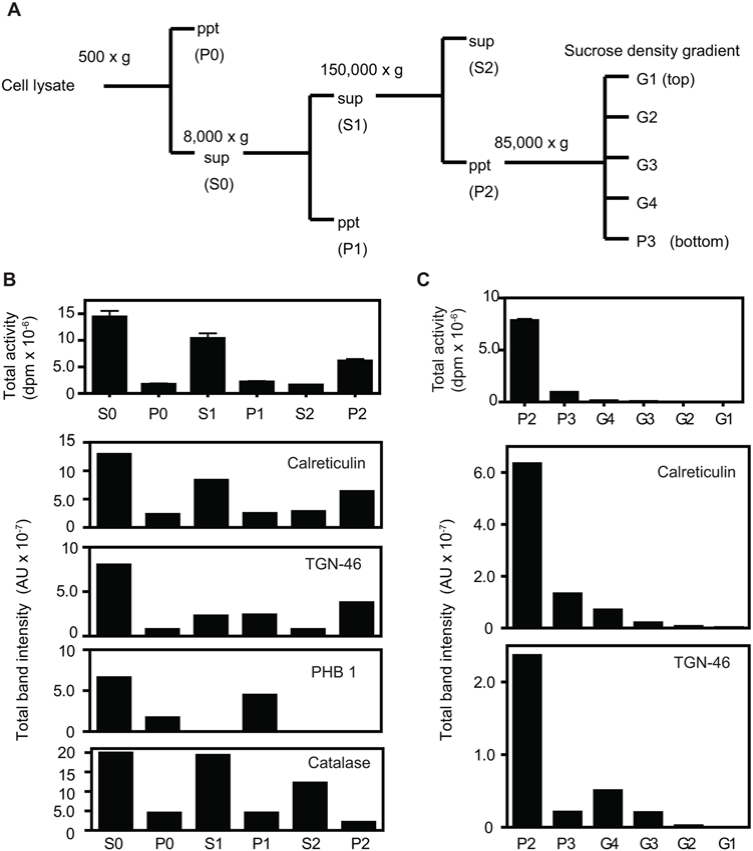
Acyltransferase activity is enriched in endoplasmic-reticulum-containing fractions. (A) Schematic representation of fractions obtained using differential centrifugation. Details of this experiment are described in Materials and Methods. “Sup” and “ppt” indicate the supernatant and pellet, respectively, obtained from each centrifugation. (B) The total acyltransferase activity in each fraction was calculated using 2.5 µg protein under standard assay conditions and correcting for the total protein in each fraction. Two and a half µg of protein from each fraction were subjected to SDS-PAGE, and analyzed by Western blotting using each subcellular marker; calreticulin, ER; TGN-46, Golgi; catalase, peroxisome; prohibitin-1, mitochondria. (C) The P2 microsomal fraction was subjected to sucrose gradient centrifugation. Two and a half µg protein (10 µg for the TGN fraction) from each fraction were subjected to SDS-PAGE and analyzed by Western blotting using antisera against proteins with known subcellular localizations. The band intensities of marker proteins were measured using an Alphaimager 3300, and the total band intensity of each marker protein in each fraction was calculated as described in the Materials and Methods. Acyltransferase samples were assayed in duplicate, and the mean and standard deviation are shown. Results are given as total dpm of octanoyltransferase activity in each fraction, and as arbitrary units of total band intensity per fraction. One of four independent fractionation experiments is shown; all gave essentially the same results.

We used subcellular fractionation to determine the location of our acyltransferase within cells. Figure 3A depicts the fractionation procedure, while Figure 3B shows that most of the acyltransferase activity (top panel) was present within the P2 microsomal fraction and colocalized with the ER and Golgi markers (calreticulin and TGN-46, respectively; quantitation of Western blots shown in panel 3B). In contrast, the expression of other organelle markers, such as PHB1 (prohibitin1; mitochondria) and catalase (peroxisomes) did not correspond to fractions with high acyltransferase content. This fractionation experiment was repeated four times with similar results. When the P2 fraction was further fractionated on a sucrose gradient, most of the acyltransferase activity was concentrated in the P3 fraction, again colocalizing with the ER marker protein calreticulin, but not with the Golgi marker TGN-46 (G4 and G3 fractions) (Figure 3C). These results indicate that the acyltransferase is localized to the endoplasmic reticulum (ER); we have therefore termed this enzyme ERAT (ER Acyl Transferase).

### Purification of ERAT

We were able to partially isolate ERAT activity using a combination of ion-exchange FPLC columns. Salt-extracted microsomal fractions were subjected first to chromatography on a Mono Q column, where activity eluted at about 150 mM NaCl. The active fractions were then loaded onto a SP-Sepharose column and eluted with sodium chloride gradient; active fractions were subjected to chromatography on a Mini-S column (Figure S1A), where activity eluted at about 300 mM NaCl. Coomassie staining of these fractions indicated a considerable decrease in protein complexity (Figure S1B). Gel filtration experiments of ion exchange-purified material demonstrated that ERAT activity eluted with an Mr between 40 to 60 kDa (Figure S1C). Mass spectroscopic analysis of the Mini-S fraction indicated enrichment in 20 different proteins (Table S1).

### ERAT Exhibits a Neutral pH Optimum and Is Stimulated by Metals

**Figure 4:**
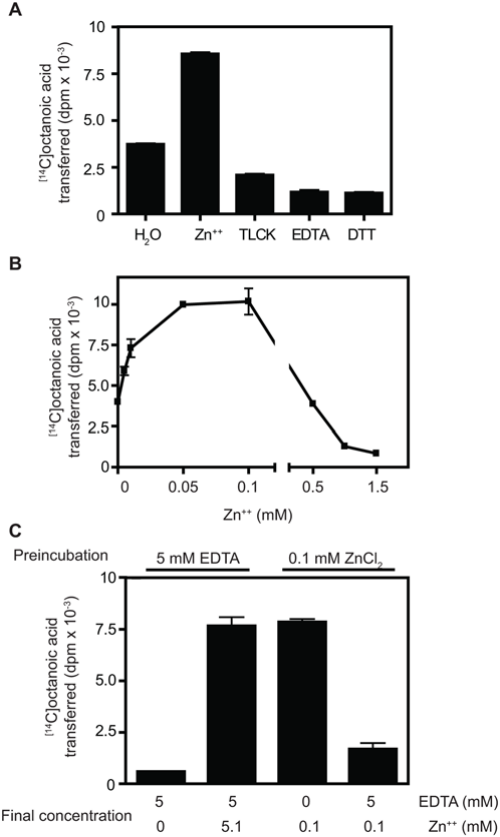
Zinc directly enhances ERAT activity. All experiments in this figure were performed with aliquots of the most active fraction from Mini-S chromatography as of acyltransferase enzyme source. To characterize the biochemical properties of ERAT, 2 µg of GAM-proghrelin were incubated for 2 h with either 0.1 mM ZnCl_2_, 5 mM EDTA, 1 mM TLCK or 5 mM DTT (A), or with the different concentrations of ZnCl_2_ indicated in (B). In order to determine whether zinc acts directly or indirectly on ERAT, GAM-proghrelin was pre-incubated with either 0.1 mM ZnCl_2_ or 5 mM EDTA (C). After this preincubation, [^14^C]octanoyl CoA was added either with or without additional zinc, as shown. Acylation reactions were then carried out at 37C for 1 h. All samples were tested in duplicate, and the mean and standard deviation are shown. Results are given as dpm of [^14^C]octanoic acid transferred to GAM-proghrelin per reaction.

To better understand the catalytic mechanism of this enzyme, reactions were carried out using different pH conditions and various ions and inhibitors using aliquots of the peak fractions from Mini-S chromatography. ZnCl_2_ significantly stimulated acyltransferase activity at low concentrations (Figure 4A, compare lanes 1 and 2), though not at higher concentrations. CoCl_2_ also slightly stimulated the enzyme activity, while other divalent metal ions did not (data not shown). Figure 4A also shows that the reducing reagent dithiothreitol (DTT) and the chelating reagent EDTA inhibited enzyme activity (the 2 h incubation at neutral pH is likely to result in DTT oxidation; we therefore cannot say with certainty whether the inhibiting species is oxidized or reduced DTT). In addition, 1 mM TLCK (tosyllysine chloromethyl ketone) produced partial inhibition (Figure 4A). The stimulation by zinc and inhibition by EDTA suggests that ERAT could be a metalloenzyme. High (millimolar) concentrations of ZnCl_2_ produced much less effective stimulation than lower concentrations (Figure 4B).

Since in addition to being stimulated by zinc, ERAT was profoundly inhibited by metal-chelating reagents, the possibility existed that the metal stimulation effect was indirect, i.e. that acyltransferase activity could require an early zinc-dependent proteolytic step. This could consist either of zinc-dependent proteolytic activation of ERAT, or a zinc-dependent proteolytic processing of the substrate (for example, to expose a putative acylation consensus sequence, similar to that seen for myristoylation). In order to investigate this idea, we asked whether zinc activation precedes the acylation step or coincides with it. Aliquots of the peak fractions from Mini-S chromatography were preincubated with GAM-proghrelin and either 0.1 mM ZnCl_2_ or 5 mM EDTA. After the preincubation period, the acylation reaction was initiated by adding [^14^C]octanoyl CoA. All reactions received the standard zinc addition at this point; some reactions also received excess EDTA. If zinc primarily acted on a putative protease reaction occurring prior to acylation, then we would expect that the addition of EDTA to the zinc-preincubated reaction would no longer result in inhibition of acylation (since the putative zinc-dependent proteolytic reaction would have already taken place). However, EDTA addition was still able to dramatically reduce acylation in a zinc-preincubated reaction (Figure 4C). These data support the idea that zinc acts directly on the acyltransferase reaction rather than on an earlier zinc-dependent proteolytic event.

**Figure 5:**
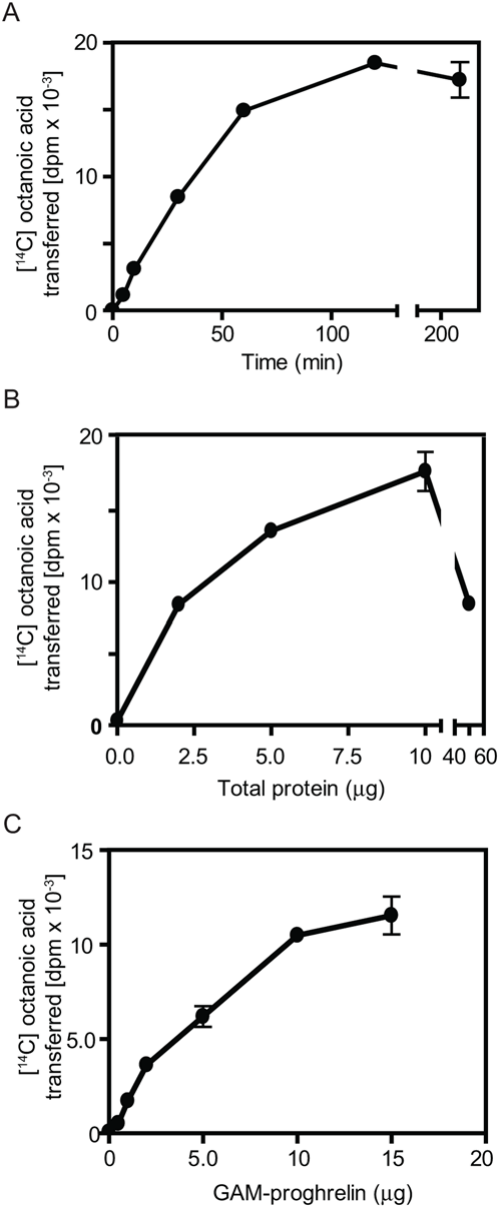
ERAT is time- and protein-dependent. Enzyme activity was determined at the indicated times (A), while the dependence of activity on the amount of HEL cell P2 protein and GAM-proghrelin is shown in (B) and (C), respectively. Samples were tested in duplicate under the standard reaction conditions, and the mean and standard deviation are shown. Results are given as dpm of [^14^C]octanoic acid transferred to GAM-proghrelin.

Under optimal conditions (50 mM NaCl and 0.1 mM ZnCl_2_ ) the transfer of [^14^C]octanoyl CoA to GAM-proghrelin increased continuously for almost 2 h (Figure 5A). The amount of [^14^C]octanoyl CoA transferred to GAM-proghrelin was dependent on the amount of HEL microsomal protein up to 10 µg; however, protein concentrations above 50 µg/reaction were inhibitory (Figure 5B). With more highly purified material from Mini-S column chromatography, the inhibitory effect at high protein concentrations was not observed (data not shown). These results support the idea that the decrease in activity is caused by other factors in the P2 microsomal fraction, possibly hydrolases. The amount of [^14^C]octanoyl CoA transferred to proghrelin was also dependent on the amount of GAM-proghrelin added to the reaction between 0 and 15 µg (Figure 5C).

**Figure 6:**
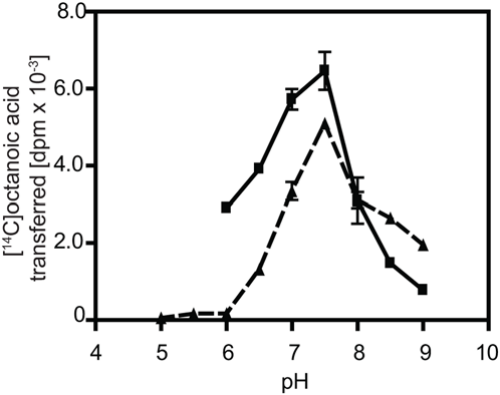
ERAT has a neutral pH optimum. Enzyme activity was determined at pHs between 5.0–9.0 under standard conditions using aliquots of active fractions from Mini-S chromatography. The indicated pH conditions were reached using either 50 mM Tris-HCl buffer *(solid line)* or 50 mM sodium phosphate buffer (*broken line*) in independent experiments. Samples were tested in duplicate at each pH point and the mean and standard deviation are shown. Results are given as the dpm of [^14^C]octanoic acid transferred to GAM-proghrelin.

Using two different buffer systems, maximal activity was obtained at neutral pH, consistent with the pH of the endoplasmic reticulum (Figure 6).

### ERAT Specificity

**Figure 7:**
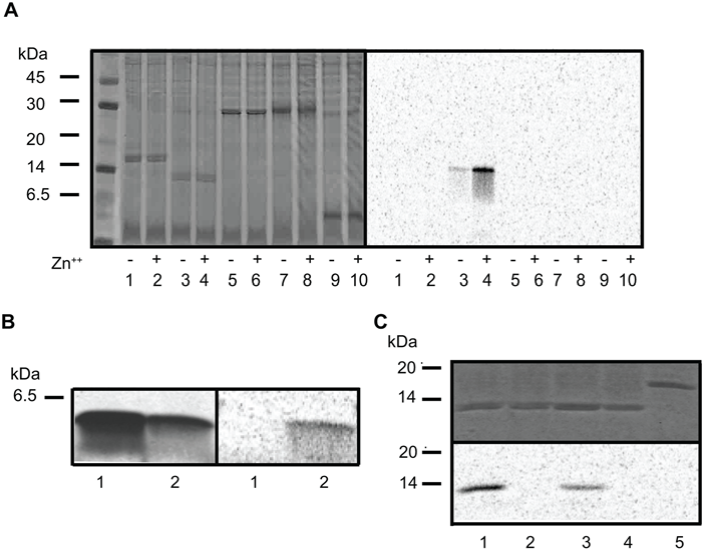
ERAT acylates GAM-proghrelin, requires certain amino acids at the N-terminus of the acylated protein, and is specific for Ser5. (A) ERAT acylates GAM-proghrelin, but not His-tagged proghrelin, nor other precursors. Two µg of each peptide precursor were incubated with HEL cell P2 protein in the presence or absence of 0.1 mM Zn^++^. *Lanes 1 and 2*, His-tagged proghrelin; *lanes 3 and 4*, GAM-proghrelin; *lanes 5 and 6*, mouse POMC; *lanes 7 and 8*, rat proenkephalin; *lanes 9 and 10*, ACTH. *Left panel*, Coomassie-stained gel of reaction mixtures showing the presence of equal quantities of each substrate; *right panel*, autoradiogram of the same gel, to identify [^14^C]octanoylated bands. (B) ERAT requires certain amino acids at the N-terminus of the acylated protein. Two µg of the peptides des-acyl ghrelin (*lane 1*) or GAM-des-acyl ghrelin (*lane 2*) were tested as potential substrates for ERAT. *Left panel*, autoradiogram of reaction mixtures to identify [^14^C]octanoylated bands; *right panel*, Coomassie-staining of the same gel to demonstrate the quantity of peptide. (C) ERAT is specific for Ser5: lack of acylation of the GAM-proghrelin mutants and preproghrelin. Acyltransferase activity was tested using GAM-proghrelin (*lane 1*), each mutant (S5A, *lane2*; S6A, *lane 3*; and S5,6A, *lane4*) and preproghrelin (*lane 5*) under the standard reaction conditions. *Lower panel*, autoradiogram to identify [^14^C]octanoylated bands; *upper panel*, Coomassie staining of the same gel in order to demonstrate the presence of equal quantities of each substrate.

Figure 7A demonstrates that two other peptide hormone precursors, POMC (proopiomelanocortin) and proenkephalin were not acylated- even though POMC is a known precursor of an acylated peptide, α-MSH. Again, His-tagged proghrelin was not acylated. This figure also demonstrates the remarkable stimulatory effect of the inclusion of 0.1 mM ZnCl_2_ on the reaction. Interestingly, a modified ghrelin peptide containing the extra amino acids GAM at the N-terminus was octanoylated, though other small peptides such as ACTH and des-acyl ghrelin itself were inactive as substrates (Figure 7A and 7B). Surprisingly, the GAM-proghrelin mutant containing a Ser6 to Ala6 mutation was octanoylated, while the Ser5 to Ala5 mutant was not (Figure 7C, lanes 2 and 3). GAM-preproghrelin and the GAM-proghrelin mutant containing Ser5, Ser6 mutations to Ala were also ineffective as substrates (Figure 7C, lanes 4 and 5). These mutagenesis data indicate that acylation is dependent on the presence of a serine residue at position 5, and that no other serine residues are acylated (cysteine is absent in preproghrelin).

### ERAT Prefers Medium-Chain Fatty Acids

**Figure 8:**
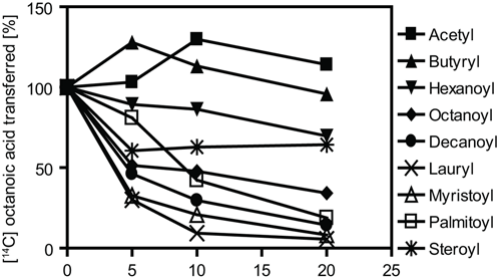
ERAT is inhibited by addition of competing acyl CoAs containing at least 8–14 carbons. Duplicate reactions containing various acylated coenzymes were incubated with 2 µg of GAM-proghrelin and the P2 fraction under standard reaction conditions. Samples were analyzed in duplicate at each point and the mean and standard deviation are shown. Results are given as the percentage of octanoic acid transfer as compared to control reactions lacking added competing acyl CoAs.

To clarify the specificity of ERAT with respect to fatty acids, eight different acyl-CoAs were added to the reaction to test competition of [^14^C]octanoic acid transfer. Activated forms of medium-chain fatty acids represented the most potent inhibitors; at 5 µM, the most inhibitory compounds were myristoyl CoA (14 carbons), lauryl CoA (12 carbons), decanoyl CoA (10 carbons), and octanoyl CoA (8 carbons). Substantial inhibition was also observed by palmitoyl and steroyl CoAs. In contrast, addition of competing fatty acyl CoAs containing small numbers of carbons, such as acetyl CoA (2 carbons), did not inhibit the ERAT reaction (Figure 8), thus implying that the enzyme cannot carry out acetylation but requires fatty acids with at least 8 carbons.

### ERAT is not N-Myristoltransferase

The fact that ERAT requires a serine residue five residues from an N-amino terminus was strongly reminiscent of the substrate specificity of N-myristoyltransferase (NMT). Indeed, mass spectrographic analysis of purified protein indicated the presence of N-myristoyltransferase in the Mini-S purified protein preparation (Table S1). In order to examine whether N-myristoyltransferase is able to octanoylate GAM-ghrelin in a zinc-dependent fashion, we obtained recombinant NMT-1 from K.E. Seaton (Hershey Center for Applied Research, Hummelstown, PA) and C.D. Smith, (University of South Carolina, Charleston, SC). As shown in Figure S2, while NMT was able to use octanoyl CoA to octanoylate GAM-ghrelin, no stimulation by zinc occurred. However, the most highly purified protein fraction, obtained following P2 fractionation, Mono-Q, SP-sepharose, Mini-S ion exchange, and gel filtration still exhibited zinc stimulation (Figure S2C). We conclude that ERAT is not NMT.

## Discussion

The field of posttranslational protein modification represents an area of increasing research interest as new protein modifying enzymes are discovered [5], [6], [7]. Still, specific posttranslational modifying enzymes remain to be identified, for example the enzymes responsible for the acetylation of the secretory peptides β-endorphin and α-MSH. These peptide modifying enzymes have been sought for over twenty years [10], [11], [12] but have never been conclusively identified, perhaps because of the large background of other cellular acyl transferases. In this report, we used a modified proghrelin substrate, GAM-proghrelin, to identify a novel zinc-stimulated acylating enzyme that is clearly not responsible for either beta endorphin or ghrelin acylation (since it does not acylate proghrelin or POMC) but most likely acylates other, as-yet-unidentified proteins. Our enzyme was highly enriched in the endoplasmic reticulum; we have therefore termed the activity ERAT.

Indeed, the specificity of ERAT was unexpected; while highly active on GAM-proghrelin, it did not acylate POMC, proenkephalin, ACTH, His-tagged proghrelin, preproghrelin or a non-His-tagged proghrelin lacking the amino-terminal tripeptide GAM. These data suggest that the amino-terminal GAM tripeptide sequence (generated by TEV cleavage of His-tagged proghrelin) is absolutely required for the acyltransferase reaction. Mutagenesis studies indicated that the preferred position for octanoylation occurs on a serine which must represent the fifth residue from the amino terminus (Ser6 was not acylated). This positioning of serine, as well as the coincidental fact that the amino terminal residue of our substrate is glycine, is strongly reminiscent of the consensus sequence for N-terminal glycine myristoylation. However, N-terminal myristoylating enzymes are neither zinc-stimulated nor EDTA-inhibited [13], nor are N-terminal acyl groups susceptible to hydroxylamine treatment, which clearly reduced serine labeling by our enzyme. We obtained a sample of N-myristoyl transferase from Drs. K.E. Seaton and C.D. Smith, used it as the enzyme source in our standard assay, and confirmed the total lack of effect of zinc on N-myristoyl transferase octanoic acid transfer (Figure S2C). Additionally, the most highly purified fraction via four sequential chromatographic steps still retains the property of zinc-dependent enzyme activity. We conclude that ERAT, though resembling myristoyl transferase in specificity, must be enzymatically distinct from this enzyme.

We also investigated the possibility that ERAT represents a member of the thiolase family. Although purified peroxisomal thiolases have previously been demonstrated to exhibit cysteine protein acylating activity [14], it is unlikely that ERAT corresponds to a peroxisomal thiolase since 1) it did not cross-react in Western blots using rabbit anti-rat thiolase antibodies (data not shown); 2) its subcellular localization did not correspond to the expression of the peroxisomal protein catalase; and 3) peroxisomal thiolases are not zinc-stimulated [14]. ERAT is likewise enzymatically dissimilar to the newly described endoplasmic reticulum-localized lysine acetyltransferases [5], which are customarily assayed in EDTA. We were unable to identify any other cellular thiolases which are known to be either zinc-stimulated or inhibited by EDTA.

Its zinc sensitivity and inhibition by EDTA also distinguishes ERAT from all other known acyltransferases. The lung enzyme rat LPCAT (acyl CoA lysophosphatidylcholine acyltransferase 1) has been reported to be Mg^++^-stimulated [15]; however, mouse LPCAT1 is known to be active in the presence of EDTA [16]. The liver enzyme LPCAT3 is a member of the MBOAT enzyme family, whose members transfer fatty acids to a wide variety of hydroxylated substrates including cholesterol, glycerol, sugars, and, more recently, proteins [17], [18]. However, like LPCAT1, LPCAT3 is apparently active in the presence of EDTA [16], [19]. In addition, primary sequence analysis indicates that the above-mentioned acyltransferases represent integral membrane proteins, while ERAT is clearly a peripheral membrane protein. For similar reasons ERAT is also unlikely to be closely related to the recently described MBOAT protein acyltransferase GOAT [6], [7]. Thus the acyltransferase described here appears to represent an enzymatic entity physically and enzymatically distinct from all other acyltransferases described to date.

Since thus far no acylation enzymes have reported to be zinc-stimulated, the question can be raised as to whether ERAT activity is associated with a zinc-dependent proteolytic event, or represents a single catalytic entity. However, preincubation of the enzyme substrate-reaction with zinc prior to addition of octanoyl CoA did not result in any loss of zinc stimulation or of EDTA inhibition during the actual acyl transfer reaction. In addition, enzyme purified using four sequential chromatographies still exhibited robust zinc stimulation and EDTA inhibition. These two results support the idea that ERAT represents a single catalytic entity, although the tight association of a metal-stimulated protease which acts in conjunction with acylation cannot definitively be ruled out.

In summary, in the work presented here we have identified a novel ER protein serine acyltransferase activity; we are currently attempting to derive sequence information for ERAT as well as to identify possible endogenous substrates.

## Materials and Methods

### Materials

The preparation of rat proenkephalin from CHO cell-conditioned medium has been described previously [20]. The preparations of mouse POMC and His-tagged human proghrelin from *E. coli* have also been described [21]. Treatment of His-tagged proghrelin with TEV protease to remove the His-tag has been described [21]; this treatment leaves a tripeptide, Gly-Ala-Met (GAM), at the N-terminus of proghrelin. ACTH and various acyl CoAs were obtained from Sigma (St. Louis, MO); des-acyl ghrelin-28 peptide was from Peptides International, Inc. (Louisville, KY); [^14^C, C-1]octanoyl CoA from Moravek Biochemicals (Brea, CA); HEL cells were obtained from the American Type Culture Collection (Rockville, MD); prohibitin1 (PHB1) antibody was from Cell Signaling Technology, Inc. (Danvers, MA): the TGN-38 monoclonal antibody and the calreticulin antibody were from Affinity BioReagents (Golden, CO); catalase antibody was from Abcam (Cambridge, MA); and the Gly-Ala-Met-ghrelin peptide (GAM-peptide) was synthesized by the Biopolymer Core at the University of Maryland-Baltimore.

### Preparation of Microsomal Fraction

HEL cells were grown in 2 L batches in RPMI 1640 medium containing 10 mM HEPES, 10% FBS, 110 µg/ml sodium pyruvate, and 2 mM glutamine for 5 days at 37C. Cells (10^8^∼10^9^ cells total) were then harvested by centrifugation and homogenized using six strokes in a Teflon-glass homogenizer in 25 ml of ice-cold 10 mM Tris-HCl buffer (pH 7.5) containing 0.25 M sucrose, and then centrifuged at 750× g for 10 min at 4C. The pellet was resuspended in 10 ml of ice-cold 10 mM Tris-HCl buffer, homogenized and centrifuged again at 750× g for 10 min at 4C to remove unbroken cells and nuclei. The supernatants were combined and subjected to additional centrifugation at 8,000× g for 20 min at 4C. The pellet was resuspended in 10 ml of ice-cold 10 mM Tris-HCl buffer, re-homogenized and centrifuged again at 8,000× g for 20 min at 4C. These supernatants were combined and subjected to centrifugation at 150,000× g for 35 min at 4C. This supernatant was then removed, and the pellet, the P2 microsomal fraction, was resuspended in 10 mM Tris-HCl, pH 7.5 containing 300 mM sucrose and 50 mM NaCl, frozen in aliquots, and used as the enzyme source. To determine the optimal extraction efficiency of acyltransferase activity, after centrifugation at 150,000× g the P2 pellet was resuspended in 10 mM Tris-HCl, pH 7.5, and 100 mM NaCl containing either 1% Triton X-100; 1 M NaCl; both; or neither. The homogenates were then centrifuged again at 150,000× g for 35 min at 4C. For the Triton-insoluble fraction, further extraction was performed with 1 M NaCl, followed by centrifugation at 150,000× g for 35 min at 4C. The supernatants were collected and then dialyzed against at least 10,000 volumes of 50 mM Tris-HCl, pH 7.5, containing 50 mM NaCl. The pellets were resuspended in the same buffer and were also dialyzed in order to remove NaCl. Dialyzed samples were frozen in aliquots and used as the enzyme source in the enzyme extraction experiment. Protein concentrations were measured in each sample using the Bio-Rad modified Bradford assay.

Enzyme preparations were diluted in buffer to achieve a final protein concentration of 0.5 mg/ml and, when present, of Triton X-100 to 0.03%, well below the expected inhibitory concentration to the acyltransferase activity (0.1% Triton X-100 results in 20% inhibition; data not shown).

### Octanoyltransferase Assay

The octanoyltransferase reaction was routinely carried out for 2 h at 37 C with 2 µg of bacterially-expressed proghrelin which had been treated with TEV protease to remove the His-tag (referred to here as “GAM-proghrelin”) and 2.5 to 10 µg of protein from the active fractions obtained from Mini-S chromatography were used. For the time course and protein dependence experiments, aliquots of the P2 microsomal fraction were used. Assays were performed in 50 µl standard reactions containing 50 mM Tris-HCl, pH 7.5, 300 mM sucrose, 0.1 mM ZnCl_2_, 50 mM NaCl, 25 nCi [^14^C, C-1]octanoyl-CoA (57 mCi/mmol). The final concentrations of GAM-proghrelin and of [^14^C]octanoyl CoA were 3.6 µM and 10 µM respectively. In order to test the presence of a presumed ester bond between octanoate and GAM-proghrelin, identical volumes of either 2 M Tris-HCl, pH 8.0, or 2 M NH_2_OH were added to reaction mixtures. [^14^C]octanoylated GAM-proghrelin was then separated from free [^14^C]octanoyl CoA on 18% polyacrylamide gels (Bio-Rad Laboratories, Inc, Hercules, CA). Following Coomassie staining and brief destaining, the gel was subjected to phosphoimaging overnight, and [^14^C]octanoylated GAM-proghrelin was visualized and quantified using a Storm phosphorimaging system and ImageQuant software (GE Healthcare, Piscataway, NJ). To confirm the presence of equal amounts of substrate in each reaction, gels were stained with Coomassie blue prior to autoradiography. To calibrate the phosphoimager, a known amount of radioactivity (5 nCi) of a mixture of four [^14^C]methylated standard proteins (GE Healthcare) was applied to gels along with the reactions. After subtraction of backgrounds for each standard band (taken from a non-radioactive portion of the scan), the densities of these four bands were summed using ImageQuant and assumed to represent 5 nCi (11000 dpm). The density of each [^14^C]octanoylated protein band was then compared to this number after similar background subtraction. At maximum enzyme activity, approximately 40% of the [^14^C]octanoic acid could be transferred to GAM-proghrelin.

### Partial Purification

All purification steps were carried out at 4C. The P2 microsomal fraction from 4 L of HEL cells was resuspended in 10 mM Tris-HCl, pH 7.5, containing 250 mM sucrose, and 1 M NaCl, and was then centrifuged at 150,000× g for 35 min at 4C. The supernatant was collected and dialyzed against buffer A (20 mM Tris-HCl, pH 8.5, 50 mM NaCl and 0.05% CHAPS), and loaded onto a Mono Q 10/100 GL anion exchange column (GE Healthcare). The column was then extensively washed with buffer A, and bound proteins were eluted with a linear gradient of 50 to 500 mM NaCl in buffer A. The sample was dialyzed against buffer B (20 mM sodium phosphate buffer, pH 6.5, 50 mM NaCl and 0.05% CHAPS), and then applied to a cation-exchange column (SP-Sepharose, GE Healthcare), pre-equilibrated with buffer B. The column was extensively washed with buffer B and bound proteins were eluted with a linear gradient of 50 to 500 mM NaCl in buffer B. The active fractions were pooled and then applied to a small cation-exchange column (Mini-S 4.6/50 PE, GE Healthcare). Bound proteins were eluted with a linear gradient of 50 to 500 mM NaCl in buffer B. The active fractions were pooled and then stored at −20C until used. A Bradford protein assay (Bio-Rad Laboratories, Inc) was performed on all active fractions obtained from column chromatography.

### Gel Filtration

For further fractionation by gel filtration column, the active fractions from Mini-S were concentrated using a Centricon fitted with a YM-10 membrane (Millipore Corporation, Bedford, MA), and then 90 µg of proteins were subjected onto a Superdex-200 10/300 GL gel filtration column (GE Healthcare). The mobile phase was 50 mM Tris-HCl, pH 7.5 150 mM NaCl and 0.05% CHAPS and the flow rate was 0.25 ml/mim. The fractions containing the ERAT activity were collected and stored at −20C until assayed.

### Peptide Mapping Using Mass Spectroscopy Analysis

In order to prepare the samples for mass spectroscopy analyses, the active fraction of mini-S column was subjected to SDS-PAGE using 12.5% acrylamide gels. The gels were stained using the SilverQuest silver staining kit (Invitrogen), and the protein bands around 30–75 kDa were excised from the gels. The excised gels were subjected to in-gel trypsin digestion. The tryptic peptides were analyzed using LC-MS/MS (Xtreme Simple nano LC system, Micro-Tech Scientific, Vista, CA) and the MS/MS spectra were searched against a customized human database (downloaded on Nov. 29, 2007 from NCBI; 88,334 sequences) using Sorcerer-SEQUEST (SageN Research, Milpitas, CA). The mass spectroscopy and database searching analyses were performed by University of Maryland Greenebaum Cancer Center Proteomics Shared Service Facility.

### Subcellular Fractionation of Microsomes

The P2 microsomal fraction was resuspended in 20 mM Tris-HCl, pH 7.5, containing 1.15 M sucrose, and then subjected to sucrose density gradient centrifugation employing a system similar to that reported by Higgens and Green [22]. The gradients were composed of 1.5 ml of P2 microsomal fraction (1.15 M sucrose in 10 mM Tris-HCl, pH 7.5), 1.5 ml of medium density solution (0.85 M sucrose in 10 mM Tris-HCl, pH 7.5) and 1.5 ml of light density solution (0.25 M sucrose in 10 mM Tris-HCl, pH 7.5). After 140 min at 85,000× g, the gradients were fractionated from the top. The total amount of acyltransferase in each fraction was determined using 2.5 µg of protein from each fraction in the standard acyltransferase reaction and then multiplying by the total protein in that fraction. In order to gain information on the subcellular localization of each marker protein, 2.5 µg of protein from each fraction (except 10 µg for TGN-46) were subjected to SDS-PAGE and Western blotting using 10% or 12.5% polyacrylamide gels, and the blots were analyzed for the location of the marker proteins TGN-46, catalase, PHB1, and calreticulin, using horseradish peroxidase-coupled second antiserum and the SuperSignal West Femto Maximum Sensitivity Substrate kit (Thermo Scientific, Rockford, IL). The band intensities of marker proteins were measured using an Alphaimager 3300 (Alpha Innotech Corporation, San Leandro, CA), and the total quantities of each marker protein were calculated multiplying the intensity for the 2.5 µg protein load (10 µg was used for TGN-46) by the total protein in each fraction, as shown in Figure 3B.

### Substrate Specificity

In order to determine the substrate specificity of the enzyme, two micrograms of several other substrates, namely ACTH, mouse POMC [21], rat proenkephalin (Ozawa, unpublished results), His-tagged proghrelin [21], des-acyl ghrelin-28 and GAM- des-acyl ghrelin-28 were used instead of GAM-proghrelin. Three mutants, namely S5A, S6A, and doubly mutated (S5A, S6A) proghrelin were generated using the Quickchange mutagenesis kit (Stratagene, La Jolla, CA) with the following primers: 5′ - CAG GGC GCC ATG GGA GCC AGC TTC CTG AGC CCT GAA CAC - 3′ and 5′ - GTG TTC AGG GCT CAG GAA GCT GGC TCC CAT GGC GCC CTG - 3′ for the S5A mutant; 5′ -GGC GCC ATG GGA TCC GCC TTC CTG AGC CCT GAA CAC CAG - 3′ and 5′ - CTG GTG TTC AGG GCT CAG GAA GGC GGA TCC CAT GGC GCC - 3′ for the S6A mutant; 5′ - CAG GGC GCC ATG GGA GCC GCC TTC CTG AGC CCT GAA CAC CAG – 3′ ; and 5′ - CTG GTG TTC AGG GCT CAG GAA GGC GGC TCC CAT GGC GCC CTG – 3′ for the double mutation. For preproghrelin (including the signal peptide), the coding region was amplified with the primers, 5′- GCG GAT CCA TGC CCT CCC CAG GGA CC -3′, and 5′- CTT AAG CTT GGT ACC GAG CTC -3′, and then inserted into the pProEx (Invitrogen, Carlsbad, CA). Each His-tagged protein was expressed in bacteria and cleaved with TEV protease, similarly to GAM-proghrelin.

To test the enzyme specificity for various acyl CoAs, competing amounts of acyl CoAs were added to the standard reaction at the concentrations indicated, and the transfer rate of [^14^C]octanoic acid to GAM-proghrelin was measured and calculated using ImageQuant and Prism4 software as described above. The pH curve was obtained using 50 mM sodium phosphate buffer for pHs 5.0–9.0 and Tris-HCl for pHs 6.0–9.0 in independent experiments with aliquots of the most active fraction obtained from Mini-S chromatography. All experiments were independently replicated at least once.

### Effect of Zinc on ERAT Activity

In order to determine whether zinc acts during acylation or at an earlier step, aliquots of the active fraction from Mini-S chromatography were preincubated with GAM-proghrelin at 37C for 30 min in the presence of either 5 mM EDTA or 0.1 mM ZnCl_2_. After the preincubation, [^14^C]octanoyl CoA was added to begin the acylation reaction. The acylation reactions were then carried out for 1 h at 37C. ZnCl_2_ and/or EDTA were added to the preincubated reactions in order to adjust the final concentrations during acylation, as shown in Figure 4. [^14^C]octanoylated GAM-proghrelin levels were then analyzed by the same procedure described above.

## Supporting Information

Figure S1 ERAT purification using Mini-S chromatography and gel filtration. Following dilution, active fractions from SP chromatography were further fractionated on a Mini-S column. Proteins were eluted with a 50–500 mM NaCl linear gradient. Ninety micrograms of protein purified by Mini-S chromatography were then loaded onto the Superdex 200 10/300 GL gel filtration column. The arrow indicates the position of the highest enzyme activity (fraction #108). The molecular markers for the gel filtration were obtained from BIO-RAD; bovine thyroglobulin (670 kDa); γ-globulin from bovine (158 kDa); chicken ovalbumin (44 kDa); and horse myoglobin (17 kDa). To identify acyltransferase activity, GAM-proghrelin was incubated with aliquots of active fractions, and [^14^C]octanoic acid transfer to GAM-proghrelin was confirmed using phosphorimaging (shown within chromatogram). Aliquots of fractions containing 10 µg protein (30 µg for Mini-S fraction) were also separated on a 12.5% polyacrylamide gel and Coomassie-stained (panel B). Lane 1, cell lysate; lane 2, P2 microsomal fraction; lane 3, salt-extracted fraction; lane 4, active fraction from Mini-S column chromatography. A zinc-stimulation experiment was also performed using peak fractions from both Mini-S and gel filtration chromatography in order to ensure that we were still following zinc-stimulated enzyme (panel D).
(9.26 MB EPS)

Figure S2 N-Myristoyltransferase can octanoylate GAM-ghrelin but is not zinc-stimulated. Octanoylation of GAM-pghrelin was examined using 0.1 µg of N-myristoyltransferase under the same reaction conditions as the ERAT reaction. The reactions were carried out with or without 0.1 mM ZnCl_2_. For the ERAT reaction, the most active Mini-S fraction was used as the enzyme source. Panels A and B show the autoradiogram and the quantities of [^14^C]octanoic acid transferred to GAM-proghrelin, respectively.
(1.09 MB EPS)

Table S1 Summary of mass spectroscopy analysis of Mini-S purified ERAT.
(0.05 MB DOC)
