# Supplementary material for: Enzymatic Characterization of a Human Acyltransferase Activity
Source: PLoS One. 2009 May 4;4(5):e5426. doi: 10.1371/journal.pone.0005426 (PMC2672172; doi:10.1371/journal.pone.0005426)
Supplement: Table S1 — Summary of mass spectroscopy analysis of Mini-S purified ERAT. (0.05 MB DOC) [file pone.0005426.s003.doc]

Table S1. Summary of mass spectroscopy analysis of Mini-S purified ERAT

| Protein name | Number of unique peptides | Total number of peptides | Molecular weight (kDa) |
| --- | --- | --- | --- |
| Moesin | 84 | 1031 | 67.8 |
| HMG-2 | 16 | 59 | 24.0 |
| N-Myristoyl CoA transferase | 10 | 43 | 56.8 |
| Hypothetical protein MGC13064 | 8 | 36 | 22.7 |
| Elva-like protein1 | 7 | 8 | 36.1 |
| U1 small nuclear ribonucleoprotein A | 5 | 13 | 31.3 |
| FLJ14805 | 4 | 13 | 44.9 |
| Nuclear protein HCC1 | 4 | 16 | 23.7 |
| FLJ23027 | 4 | 7 | 42.9 |
| NSAP1 protein | 4 | 10 | 62.7 |
| Moesin/anaplastic lymphoma kinase fusion protein | 3 | 4 | 61.9 |
| 28 kDa heat- and acid-stable phosphoprotein | 3 | 11 | 20.6 |
| FRAP-related protein | 2 | 16 | 30.1 |
| Acetolactate synthase homolog | 2 | 5 | 67.9 |
| NF-AT 45k chain | 2 | 3 | 44.7 |
| EBP1 | 2 | 7 | 38.1 |
| Possible global transcription activator SNF211 | 2 | 3 | 88.6 |
| Hypothetical protein DKFZp762H157.1 | 2 | 3 | 69.4 |
| Thioredoxin peroxidase | 2 | 5 | 22.3 |
| Kinesin-like DNA-binding protein | 1 | 9 | 73.3 |
